# Comparing proton pump inhibitors with histamin-2 receptor blockers regarding the risk of osteoporotic fractures: a nested case-control study of more than 350,000 Korean patients with GERD and peptic ulcer disease

**DOI:** 10.1186/s12877-020-01794-3

**Published:** 2020-10-15

**Authors:** Joo-Hyun Park, Jessie Lee, Su-Yeon Yu, Jin-Hyung Jung, Kyungdo Han, Do-Hoon Kim, Jinnie Rhee

**Affiliations:** 1 Department of Family Medicine, Korea University Ansan Hospital, Korea University College of Medicine, 123, Jeokgeum-ro, Danwon-gu, Ansan-si, Gyeonggi-do 15355 Republic of Korea; 2 National Evidence-based Healthcare Collaborating Agency, Seoul, Republic of Korea; 3 Department of Biostatics, Catholic University College of Medicine, Seoul, Republic of Korea; 4 Department of Statistics and Actuarial Science, Soongsil University, Seoul, Republic of Korea

**Keywords:** Proton-pump inhibitor, Osteoporosis, Fracture, Peptic ulcer disease, Gastroesophageal reflux disease

## Abstract

**Background:**

Patients with peptic ulcer disease (PUD) and gastroesophageal reflux disease (GERD) are more likely to receive long-term therapy with proton pump inhibitors (PPIs). This study aimed to investigate the risk of osteoporotic fractures in PPI users compared to histamine-2 receptor antagonist (H2RA) users and the association between fractures and the duration and regular use of PPI.

**Methods:**

A population-based, nationwide nested case-control study from January 2006 to December 2015 was performed using Korean National Health Insurance Service claims data. We included patients ≥50 years of age, without previous fractures, newly prescribed with PPI or H2RA, and diagnosed with PUD or GERD from 2006 to 2015. Patients with osteoporotic fracture (*n* = 59,240) were matched with the non-fracture control group (*n* = 296,200) at a 1:5 ratio based on sex, age, cohort entry date, follow-up duration, and bisphosphonate use. The osteoporotic fractures were defined using the diagnostic codes of claims data (M80, M81, M82, M484, M485, S220, S221, S320, S327, S422, S423, S525, S526, S72).

**Results:**

The higher the cumulative use of PPIs, the higher the osteoporotic fracture risk (*P* for trend < 0.001). The risk of osteoporotic fracture in the patients whose cumulative use of PPI was more than 1 year was higher than that of others (OR: 1.42, 95% CI: 1.32–1.52). Patients who regularly used PPI in the recent 1 year had a higher risk of osteoporotic fracture than exclusive H2RA users (OR: 1.37, 95% CI: 1.26–1.50).

**Conclusions:**

The risk of osteoporotic fracture increased with the duration of PPI use, especially when PPI was used for ≥1 year and regularly in the recent 1 year.

## Background

Gastroesophageal reflux disease (GERD) and peptic ulcer disease (PUD) are very common disorders with a long disease course. Therefore, proton pump inhibitors (PPIs), which are used for the treatment of GERD and PUD, are among the most frequently prescribed drugs for long-term use worldwide [1, 2]. In general, PPIs are considered relatively safe drugs with few short-term adverse effects; however, an increasing number of studies have reported on adverse effects associated with long-term use [3]. As one of them, it is suggested that reducing calcium absorption through the inhibition of gastric acid secretion by using PPI may cause an osteoporotic fracture [4–8].

Osteoporotic fractures are a major health problem worldwide and are expected to further increase in incidence with increases in life expectancy [9, 10]. Osteoporotic fractures are a major cause of increased mortality [11], increased number of hospitalizations [12, 13], high health-care costs, and reduced quality of life [14]. As PPIs are effective and widely used drugs, it is necessary to investigate the duration and patterns of their use, as well as what extent of PPI use is associated with osteoporotic fractures.

Prior studies that reported that PPIs are associated with osteoporotic fractures yielded inconsistent results on the duration and use patterns of PPIs, which influence the osteoporotic fracture risk. Most studies have reported that any use [15–24] or current use [25–27] of PPIs increases the osteoporotic fracture risk. However, the association between the duration of PPI use and osteoporotic fracture has been inconsistently reported, with some studies showing a relation [21] and others reporting no relation [27, 28]. In addition, previous studies were mostly performed other than Asian countries. Therefore, studies based on Asian population are needed because there are genetic and ethnic differences in the pharmacological action of PPI [29], in bone mineral density [30], and in the prevalence of osteoporotic fractures.

Therefore, this study aimed to investigate the risk of osteoporotic fractures associated with PPI use compared to exclusive H2RA use, according to the duration of use and regular use of PPIs. H2RA users are the desirable comparator of PPI users because H2RA users are more likely to have underlying gastrointestinal disorders similar to PPI users, and H2RA’s gastric secretion inhibition is weaker than PPI.

## Methods

### Data source

We used the Korean National Health Insurance Service (NHIS) claim data and the biannual regular health examination data. South Korea has a mandatory social health insurance system covering approximately 97% of the entire population. Almost all medical data, including diagnostic codes, procedures, prescription drugs, and personal information, are included in the NHIS database. The NHIS also conducts a standardized national health screening program every 2 years for all insured persons aged over 40 years and every year for employee subscribers aged 20 years or older. National health screening data include anthropometric measurements, laboratory tests, and social history.

Patients with the International Classification of Diseases (ICD)-10 codes for PUD or GERD (K21, K25, K26, K27, and K28) and a prescription history of PPIs or histamine-2 receptor antagonists (H2RAs) from January 1, 2006 to December 31, 2015 were extracted. Subsequently, information on demographic characteristics and lifestyle or health behavior information (age, sex, residence, income level, height, weight, body mass index, smoking, alcohol drinking, exercise, etc.) were extracted.

All personal identification numbers have been encrypted by converting them into scrambled numbers before data processing, to comply with the privacy rules of the health insurance transfer and privacy laws. Therefore, the use of anonymized information exempted this study from the need for written consent. This study was conducted in accordance with the tenets of the Helsinki Declaration. All research procedures and ethical aspects were approved by the Institutional Review Board of the National Evidence-based Healthcare Collaborating Agency (approval no. 17–011).

### Study design and patient selection

We included patients with GERD and PUD, who were prescribed PPI or H2RA at least once with this diagnosis. We did not include non-users of gastric acid inhibitor, who have never used PPI or H2RA during the observation period. Comparing PPI users with non-users of a gastric acid inhibitor can lead to an overestimation of PPI risks. Individuals who have never used PPI or H2RA during the observation period are likely to be in relatively good health and may not have peptic ulcer disease or gastritis, reducing calcium absorption and affecting fracture risk. Therefore, we evaluated the risk of osteoporotic fractures associated with PPI use compared to H2RA use to avoid overestimating the PPI effect. The cohort entry date was defined as the date when the first drug (PPI or H2RA) was prescribed. We defined the case group as patients who developed osteoporotic fractures during the follow-up period and the control group as those who did not develop osteoporotic fractures. A nested case-control study was performed to examine the risk of osteoporotic fracture between the PPI use group and the H2RA use group.

A total of 35,520,188 patients were taking either PPI or H2RA for PUD and GERD from January 1, 2006 to December 31, 2015. Only new users who did not have a history of being prescribed with PPI or H2RA within the first year of cohort entry were included. The exclusion criteria were as follows: age < 50 years on cohort entry; medical service use because of cancer (ICD-10 code: C, T), acquired immunodeficiency syndrome (B20–24), or osteoporosis (M80, 81) within 1 year before entering the cohort; or a diagnosis of osteoporotic fracture (spine: S220, 221, 320, 327, M484, 485; proximal humerus: S422, 423; femur: S72; distal radius: S525, 526) within 1 year before cohort entry. Patients with missing values in the matching variables were also excluded. To use the variables included in the health screening data as covariates, those who did not undergo a national health screening were excluded. After the exclusions, 2,388,137 patients remained.

The outcome index (osteoporotic fracture) was defined using the codes requested from the health insurance claims data. First, patients with osteoporosis with pathological fracture were selected (disease classification code: M80). Second, patients who had a diagnosis of osteoporosis (M81, 82) and experienced an osteoporotic fracture after study entry were collected. Osteoporotic fractures were defined as fractures of 4 parts: spine (S220, 221, 320, 327, M484, 485), proximal humerus (S422, 423), femur (S72), and distal radius (S525, 526). Patients in whom osteoporosis was diagnosed before osteoporotic fracture or within 90 days after osteoporotic fracture were defined as the osteoporotic fracture group. The patients who had osteoporotic fractures during the follow-up period (from cohort entry to 2015) was 78,465.

**Fig. 1:**
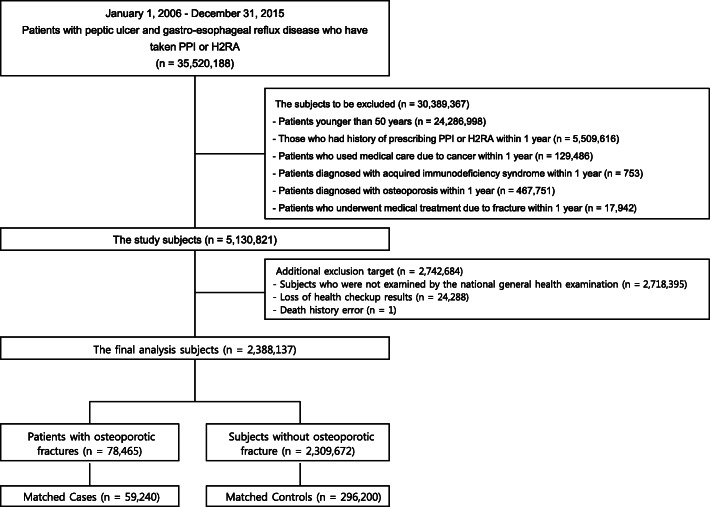
Flowchart of patient selection. PPI, proton pump inhibitor; H2RA, histamine-2 receptor antagonist

The index date was the time of osteoporotic fracture. When the osteoporotic fracture occurred more than twice during the study period, the time of the first fracture was used in the analysis. Corresponding to the osteoporotic fracture group, the control group was selected, through 1:5 matching according to sex, age group with a 5-year interval, and use of bisphosphonates, among patients who did not develop osteoporotic fractures within the same cohort entry and follow-up period. Finally, we had 59,240 matched cases and 296,200 controls. The process of selecting and excluding patients for the final analysis is shown in Fig. 1.

### Definition of PPI exposure and covariates

The criteria for PPI use were ‘any use’, ‘duration of use’, and ‘regular use’. We defined any use of PPI as being prescribed PPI at least once during the study period. We also defined PPI duration as the cumulative days of use calculated by adding the prescriptions days regardless of continuity. The cumulative days of PPI were categorized as < 30, 30–59, 60–89, 90–179, 180–364, and ≥ 365 days. The recent 1 year prior to osteoporotic fracture was divided into four quarters. We identified the number of quarters with PPI use from one to four. The use of PPI over four quarters was defined as “regular use.”

We considered various covariates associated with the development of osteoporotic fractures due to the use of PPI. These include socioeconomic and demographic factors such as sex, age, residence area, income, and anthropometric and lifestyle factors such as body mass index, drinking, smoking, and exercise. We obtained information on health behaviors through a standardized self- administered questionnaire collected on the day of the national health screenings. According to smoking status, subjects were classified into non-smokers, former smokers, and current smokers. Heavy alcohol drinkers were defined as those who consumed ≥30 g/day of alcohol. Physical activity was classified as follows according to the frequency of ≥20 min of strenuous exercise (none, ≤ four times per week, and ≥ five times per week). The national health screenings also included measurements of body size. Body mass index was calculated by dividing the body weight by the square of the height (kg/m2). Income levels were classified into quintile groups using the NHIS qualification data.

These covariates also consisted of concomitant diseases and concomitant medications that could directly or indirectly affect osteoporotic fracture development. The use of bisphosphonates, glucocorticoids, anticonvulsants, hormone replacement therapy, warfarin, heparin, antacids, selective serotonin reuptake inhibitors, benzodiazepines, and tricyclic antidepressants was considered concomitant medication use. All were defined using the drug code (main ingredient code). Concomitant diseases were defined using the ICD-10 codes and included chronic obstructive pulmonary disease (J44, 45), testicular dysfunction (E29.1), hypothalamic dysfunction (E23.0), hyperthyroidism (E05), hyperparathyroidism (E21), Cushing’s syndrome (E24), hyperprolactinemia (E22.1), vitamin D deficiency (E55.9), idiopathic hypercalciuria (E83.5), diabetes (E11–14 and diabetic agents), anorexia nervosa (F50.0, 50.1), systemic lupus erythematous (M32), hypertension (I10–13, I15, and use of antihypertensive agents), intestinal absorption disorder (K90), inflammatory bowel disease (K50, 51), chronic kidney disease (N18), and secondary amenorrhea (N91.1). Another variable considered as a covariate was the Charlson Comorbidity Index (CCI). Comorbidities and medications used were defined as at least one claim for the relevant drug code and diagnostic code for the period from 1 year before the date of cohort entry to the end of follow-up.

### Statistical analysis

The categorical variables were presented as frequencies and percentages of the patients. For continuous variables, means and standard deviations were presented.

Conditional logistic regression models were used to estimate odds ratios (ORs) and 95% confidence intervals (CIs) to investigate the association of PPI use and osteoporotic fracture compared to the exclusive H2RA use. The crude model was not adjusted for covariates, and the full-adjusted model included adjustments for age, sex, body mass index, alcohol drinking, smoking, exercise, concomitant medications, and diseases as covariates. Subgroup analyses were conducted by sex and age. Interaction tests were performed to determine whether the associations differed by sex and age. We also calculated *p* values for linear trends of osteoporotic fracture risk according to the increase in the cumulative duration and the regular use of PPI. Two-sided *P*-values < 0.05 were considered statistically significant. All statistical analyses were performed using SAS software (version 9.2; SAS Institute, Cary, NC, USA).

## Results

The mean patient age was 64.8 ± 8.0 years in the osteoporotic fracture group and 64.6 ± 8.1 years in the non- osteoporotic fracture group. Their median duration of PPI use was 30 (interquartile range, 14–74) days. We included 78.0% female patients, and the proportion of bisphosphonate use was 75.9% in both groups (Table 1).

**Table 1:** Demographic status and comorbidity in the osteoporotic fracture group and the non-osteoporotic fracture group

Variables	Osteoporotic fracture group (cases)		Non-osteoporotic fracture group (controls)		*P* value
	(*n* = 59,240)		(*n* = 296,200)		
Cohort entry year, n (%)					1.000
2006	29,139	(49.2)	145,695	(49.2)	
2007	13,356	(22.6)	66,780	(22.6)	
2008	7103	(12.0)	35,515	(12.0)	
2009	4450	(7.5)	22,250	(7.5)	
2010	2511	(4.2)	12,555	(4.2)	
2011	1378	(2.3)	6890	(2.3)	
2012	790	(1.3)	3950	(1.3)	
2013	364	(0.6)	1820	(0.6)	
2014	137	(0.2)	685	(0.2)	
2015	12	0.0	60	0.0	
Demographic characteristics					
Female sex, n (%)	46,213	(78.0)	231,065	(78.0)	1.000
Age, mean (SD), years	64.8 (8.0)		64.6 (8.1)		< 0.001
Residence, n (%)					< 0.001
Urban (large city)	30,466	(51.5)	165,016	(55.7)	
Urban (small city)	17,374	(29.3)	85,554	(28.9)	
Rural	11,373	(19.2)	45,575	(15.4)	
Household income, n (%)					< 0.001
Lowest quintile	13,252	(22.4)	64,122	(21.7)	
Health checkup/ structured questionnaire					
Height, median (IQR), cm	155 (150–160)		154 (150–160)		< 0.001
Weight, median (IQR), kg	58 (52–64)		57 (51–63)		< 0.001
BMI, median (IQR), kg/m^2^	24.0 (22.1–26.1)		23.7 (21.6–25.9)		< 0.001
BMI, n (%), kg/m^2^					< 0.001
< 18.5	2454	(4.1)	7809	(2.6)	
18.5–23	21,805	(36.8)	99,135	(33.5)	
23–25	14,959	(25.3)	79,124	(26.7)	
25–30	17,929	(30.3)	98,833	(33.4)	
≥ 30	2093	(3.5)	11,299	(3.8)	
Current smoking, n (%)	5852	(9.9)	24,306	(8.2)	< 0.001
Current alcohol drinking, n (%)	11,669	(19.7)	59,453	(20.1)	0.038
Regular exercise, n (%)	20,962	(35.4)	118,472	(40.0)	< 0.001
Concurrent disease/drug use, n (%)					
Bisphosphonates	44,949	(75.9)	224,745	(75.9)	1.000
Glucocorticoids	48,134	(81.3)	219,574	(74.1)	< 0.001
Anticonvulsants	14,735	(24.9)	49,906	(16.9)	< 0.001
HRT	6061	(10.2)	21,707	(7.3)	< 0.001
Warfarin	1114	(1.9)	3590	(1.2)	< 0.001
Heparin	7193	(12.1)	20,023	(6.8)	< 0.001
Antacids	48,175	(81.3)	215,413	(72.7)	< 0.001
SSRIs	4605	(7.8)	15,323	(5.2)	< 0.001
Benzodiazepines	46,995	(79.3)	207,468	(70.0)	< 0.001
TCAs	11,024	(18.6)	38,293	(12.9)	< 0.001
Rheumatoid arthritis	9994	(16.9)	33,677	(11.4)	< 0.001
Hyperthyroidism	4023	(6.8)	15,451	(5.2)	< 0.001
Chronic kidney disease	994	(1.7)	2990	(1.0)	< 0.001
COPD	19,700	(33.3)	84,921	(28.7)	< 0.001
Testicular dysfunction	29	(0.1)	111	0.0	0.199
Pituitary dysfunction	75	(0.1)	167	(0.1)	< 0.001
Hyperparathyroidism	302	(0.5)	620	(0.2)	< 0.001
Cushing’s syndrome	254	(0.4)	522	(0.2)	< 0.001
Hyperprolactinemia	35	(0.1)	119	0.0	0.044
Vitamin D deficiency	575	(1.0)	1392	(0.5)	< 0.001
Idiopathic hypercalciuria	1943	(3.3)	5058	(1.7)	< 0.001
Diabetes mellitus	9692	(16.4)	42,790	(14.5)	< 0.001
Intestinal absorption disorder	44	(0.1)	192	(0.1)	0.415
Chronic liver disease	5775	(9.8)	22,379	(7.6)	< 0.001
Anorexia nervosa	115	(0.2)	349	(0.1)	< 0.001
Systemic erythema lupus	183	(0.3)	508	(0.2)	< 0.001
Inflammatory bowel disease	409	(0.7)	1661	(0.6)	0.000
Secondary amenorrhea	16	0.0	133	0.0	0.052
Hypertensive disease	27,839	(47.0)	132,871	(44.9)	< 0.001
Charlson comorbidity index					< 0.001
0	19,228	(32.5)	107,655	(36.4)	
1	18,411	(31.1)	94,854	(32.0)	
2	10,021	(16.9)	47,437	(16.0)	
≥ 3	11,580	(19.6)	46,254	(15.6)	
Follow-up period, median (IQR), years	3.6 (1.8–5.7)		3.6 (1.8–5.7)		1.000

Data were presented as n (%) or mean ± standard deviation

Values are presented as mean (standard deviation, SD), number (percentage) or median (interquartile range, IQR)

PPI use was defined as having been prescribed PPI at least once during the follow-up period

Concurrent diseases were defined using the ICD-10 codes and included chronic obstructive pulmonary disease (J44, 45), testicular dysfunction (E29.1), hypothalamic dysfunction (E23.0), hyperthyroidism (E05), hyperparathyroidism (E21), Cushing’s syndrome (E24), hyperprolactinemia (E22.1), vitamin D deficiency (E55.9), idiopathic hypercalciuria (E83.5), diabetes (E11–14 and diabetic agents), anorexia nervosa (F50.0, 50.1), systemic lupus erythematous (M32), hypertension (I10–13, I15, and use of antihypertensive agents), intestinal absorption disorder (K90), inflammatory bowel disease (K50, 51), chronic kidney disease (N18), and secondary amenorrhea (N91.1)

Comorbidities and medications used were defined as at least one claim for the relevant drug code (main ingredient code) and diagnostic code during the period ranging between 1 year prior to the cohort entry date and end of follow-up

*Abbreviations*: *BMI* body mass index, *HRT* hormone replacement therapy, *SSRIs* selective serotonin reuptake inhibitors, *TCAs* tricyclic antidepressants, *COPD* chronic obstructive pulmonary disease

The OR of osteoporotic fracture of PPI users was 1.11 (95% CI: 1.08–1.13) compared to that of exclusive H2RA users in the full-adjusted model. The risk of osteoporotic fracture tended to increase with the cumulative use of PPI (*P* for trend < 0.001). The risk of osteoporotic fracture in the patients whose cumulative use of PPI was more than 1 year was higher than that of others (OR: 1.42, 95% CI: 1.32–1.52),(Table 2).

**Table 2:** Odds ratios and 95% confidence intervals for the risk of osteoporotic fracture associated with PPI use compared to exclusive H2RA use

	Osteoporotic fracture				OR (95% CI)	
	Cases		Controls			
	n	(%)	n	(%)	Crude	Adjusted^a^
Any use						
H2RA use	35,929	(61)	192,458	(65)	1 (Reference)	1 (Reference)
PPI use	23,311	(39)	103,742	(35)	1.24 (1.22–1.27)	1.11 (1.08–1.13)
Duration of use						
H2RA use	35,929	(61)	192,458	(65)	1 (Reference)	1 (Reference)
< 30 days	10,715	(17)	51,337	(18)	1.15 (1.13–1.18)	1.08 (1.06–1.11)
30–59 days	4773	(7)	21,788	(8)	1.22 (1.18–1.26)	1.09 (1.05–1.13)
60–89 days	2405	(4)	10,408	(4)	1.29 (1.23–1.35)	1.11 (1.06–1.17)
90–179 days	2747	(4)	11,249	(5)	1.37 (1.31–1.44)	1.13 (1.08–1.19)
180–364 days	1505	(2)	5417	(3)	1.57 (1.48–1.67)	1.18 (1.11–1.26)
≥ 365 days	1166	(1)	3543	(2)	1.88 (1.75–2.01)	1.42 (1.32–1.52)
P for trend					< 0.001	< 0.001
Recent use^b^						
No recent use	53,019	(90)	274,084	(93)	1 (Reference)	1 (Reference)
One quarter	3812	(6)	14,123	(5)	1.41 (1.35–1.46)	1.24 (1.19–1.29)
Two quarters	1156	(2)	4119	(1)	1.47 (1.37–1.57)	1.19 (1.11–1.27)
Three quarters	564	(1)	1793	(1)	1.64 (1.49–1.81)	1.32 (1.20–1.46)
All quarters (regular use)	689	(1)	2081	(1)	1.73 (1.59–1.89)	1.37 (1.26–1.50)
P for trend					< 0.001	< 0.001

PPI use was defined as having been prescribed PPI at least once during the follow-up period

Concomitant diseases were defined using the ICD-10 codes and included chronic obstructive pulmonary disease (J44, 45), testicular dysfunction (E29.1), hypothalamic dysfunction (E23.0), hyperthyroidism (E05), hyperparathyroidism (E21), Cushing’s syndrome (E24), hyperprolactinemia (E22.1), vitamin D deficiency (E55.9), idiopathic hypercalciuria (E83.5), diabetes (E11–14 and diabetic agents), anorexia nervosa (F50.0, 50.1), systemic lupus erythematous (M32), hypertension (I10–13, I15, and use of antihypertensive agents), intestinal absorption disorder (K90), inflammatory bowel disease (K50, 51), chronic kidney disease (N18), and secondary amenorrhea (N91.1)

*Abbreviations*: *H2RA* histamine-2 receptor antagonist, *PPI* proton pump inhibitor, *Ref.* reference

^a^Adjusted for age, sex, body mass index, alcohol drinking, smoking, physical activity, bisphosphonates, glucocorticoids, anticonvulsants, hormone replacement therapy, warfarin, heparin, antacids, selective serotonin reuptake inhibitors, benzodiazepines, tricyclic antidepressants, diabetes mellitus, chronic obstructive pulmonary disease, hypothyroidism, hypopituitarism, hyperparathyroidism, Cushing’s syndrome, hyperprolactinemia, vitamin D deficiency, idiopathic hypercalcemia, intestinal absorption disorder, chronic liver disease, rheumatoid arthritis, hyperthyroidism, chronic kidney disease, chronic obstructive pulmonary disease, anorexia nervosa, systemic lupus erythematosus, inflammatory bowel disease, secondary amenorrhea, and hypertensive disease

^b^The number of quarters with PPI use during the year prior to fracture was identified. The use of PPI over all quarters was defined as ‘regular use’

We also analyzed the risk of osteoporotic fracture according to recent regular use of PPI. The results showed that the risk of osteoporotic fracture increased as the number of quarters of PPI use during the year prior to osteoporotic fracture increased (*P* for trend < 0.001). Patients who used PPI on a regular basis in the recent 1 year had a higher risk of osteoporotic fracture than exclusive H2RA users (OR: 1.37, 95% CI: 1.26–1.50).

**Fig. 2:**
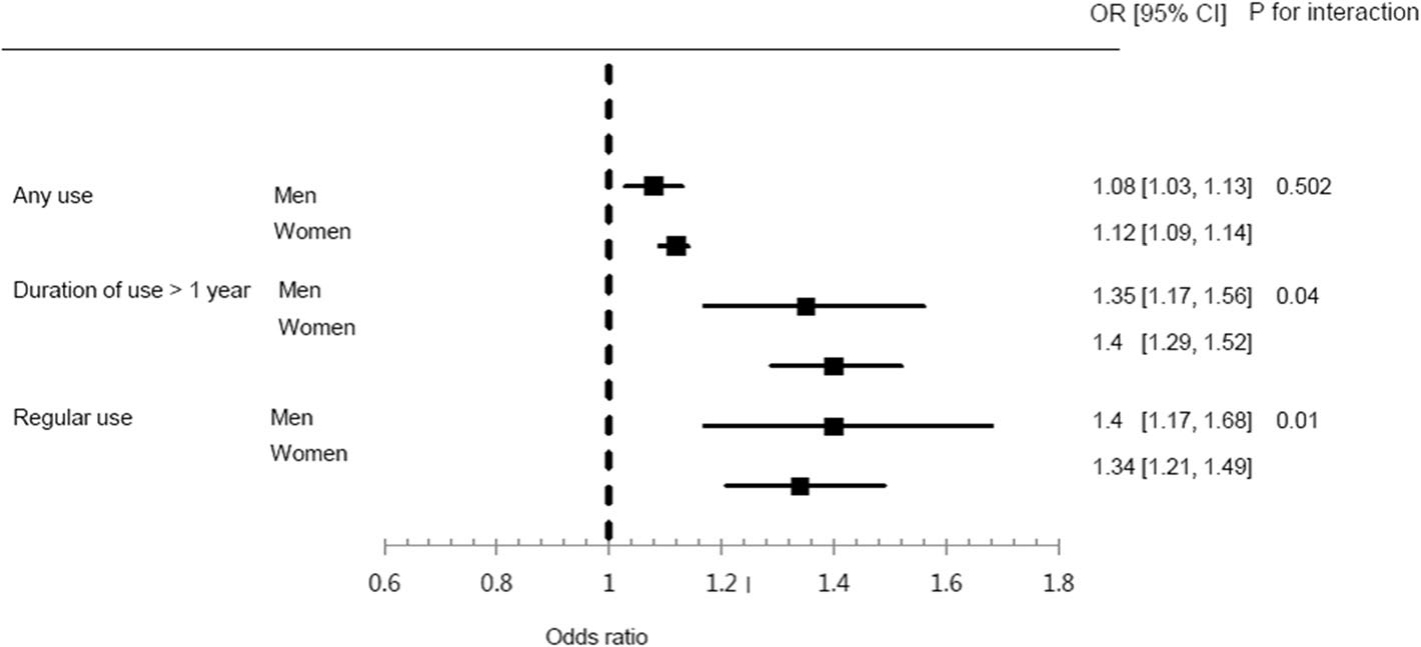
Odds ratios and 95% confidence intervals for the risk of osteoporotic fracture associated with PPI use compared to exclusive H2RA use according to sex. PPI, proton pump inhibitor; H2RA, histamine-2 receptor antagonist

Figure 2 shows the results of subgroup analysis by sex. After adjusting for all covariates, both men and women had statistically significant results with regard to the risk of osteoporotic fracture, for any use, ≥ 1 year of use, and regular use. In both men and women, the risk of osteoporotic fracture increased as the duration of use increased and as regular use increased (*P* for trend < 0.001, for all). The *P*-value for interaction was 0.502, 0.039, and 0.006 for any use, duration of use, and regular use, respectively. The effect of the duration of PPI use and regular use on the osteoporotic fracture risk differed according to sex.

**Fig. 3:**
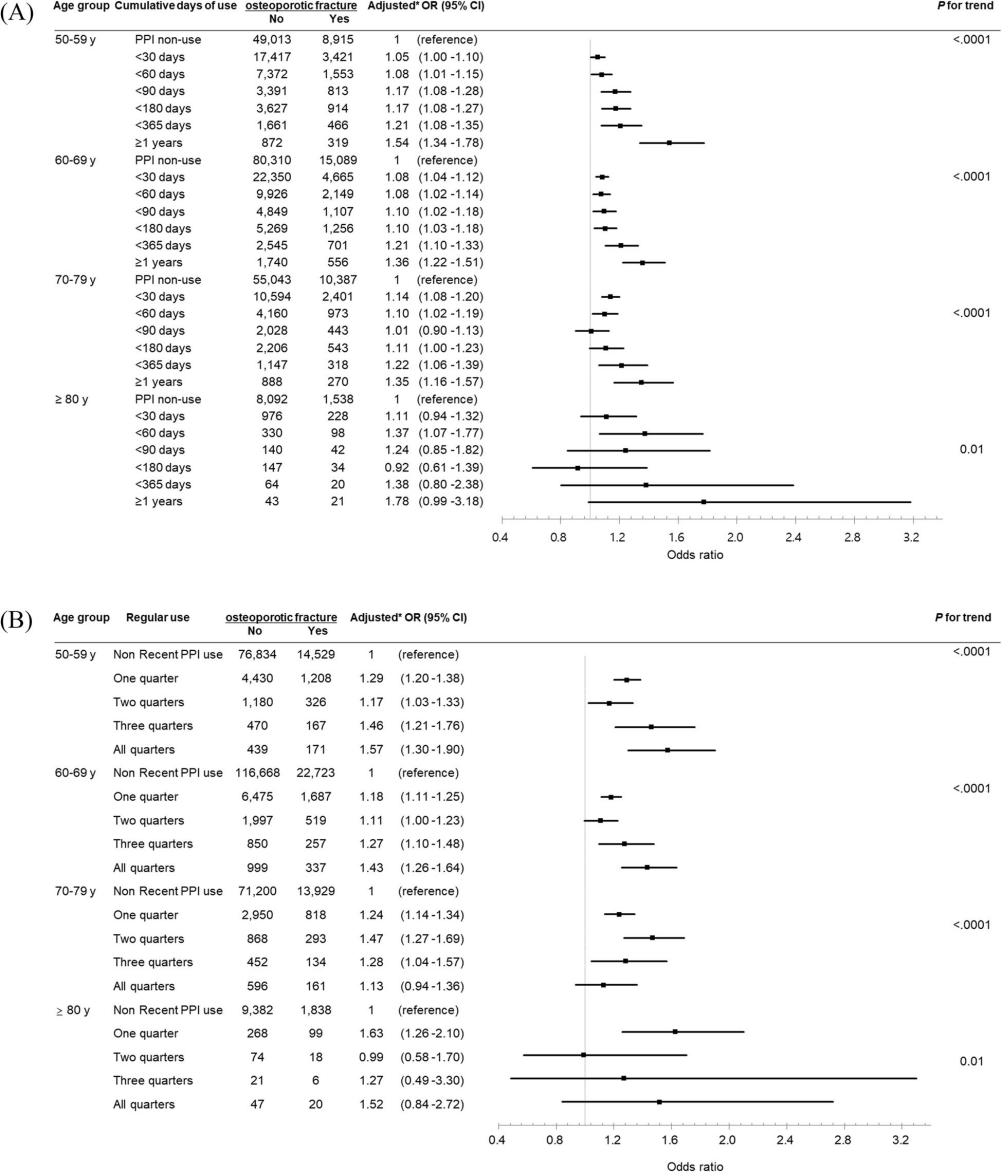
Odds ratios and 95% confidence intervals for the risk of osteoporotic fracture associated with PPI use compared to exclusive H2RA use according to age. **a** Cumulative days of use, **b** Regular use. PPI, proton pump inhibitor; H2RA, histamine-2 receptor antagonist

Figure 3 shows the duration of PPI use and regular use and the risk of osteoporotic fracture according to age group. The risk of osteoporotic fracture associated with any PPI use increased with age (all *P* for trend < 0.05). In all age groups, the risk of osteoporotic fracture increased with an increasing cumulative day of PPI use (*p* for trend: 50–79 years of age, < 0.001; ≥80 years of age = 0.01). When using PPI for more than 1 year, the osteoporotic fracture risk was highest in people over 80, but it was not statistically significant (OR, 1.78; 95% CI, 0.99–3.18). In the 50s and 60s age groups, the osteoporotic fracture risk increased with regular use (*p* for trend < 0.001).

## Discussion

The purpose of this study was to investigate the risk of osteoporotic fracture according to the use duration and regular use of PPIs by using the Korean NHIS data. In both men and women, the PPI use was significantly associated with increased risk of osteoporotic fractures, mainly when PPI was used for ≥ 1 year (adjusted OR: 1.42) and regularly for the recent 1 year (adjusted OR: 1.37). The risk of osteoporotic fracture increased by increasing the duration of PPI use and regular use.

### Interpretation and comparison with other studies

Our results are consistent with those of previous studies suggesting that PPIs are associated with osteoporotic fracture. Yang et al. reported that the use of PPI for > 1 year in patients aged ≥50 years was associated with the risk of hip fracture and that the longer the duration of treatment, the greater the association with fracture [5]. A study in Denmark reported that the use of PPI was associated with all osteoporotic fractures, including hip and vertebral fractures, within 1 year [15]. In a Canadian study, PPI exposure for > 7 years was associated with osteoporotic fracture, and there was no significant association between the use of PPI for ≤6 years and the risk of osteoporotic fracture [31]. They did not adjust the major risk factors for fracture, such as low body mass index, alcohol consumption, and smoking, so their results may differ from our study results. Corley et al. reported that the use of PPI in adults aged > 18 years was associated with an increased risk of hip fracture, and suggested that the risk of osteoporotic fracture was reduced when the drug was discontinued [6]. In a recent study in the United States, the risk of hip fracture in men aged > 45 years has been reported to increase with longer use of PPI [21]. Another study reported that PPI use increases the risk of osteoporotic fracture in elderly people aged > 65 years [20]. It has also been reported that PPI is associated with fractures in hemodialysis patients at high risk of fracture [24].

Most of the studies reported the existence of an association between PPI use and osteoporotic fracture risk. However, it was difficult to make definite conclusions because the target country, target patient group, definition of PPI use, fracture site, and covariates varied across different studies. In many studies, information on medication use was dependent on the patients’ memory recall. Further, most of the studies were Western studies and only a few were reported in Asia. A study in Taiwan indicated that the use of > 29 defined daily doses of PPI was associated with hip fracture development [32]. Asian studies are important because Asian people differ from Westerners in bone mineral density, osteoporotic fracture prevalence [30]. Also, it has been reported that the pharmacodynamics of PPI differ between Asian and Western populations because many Asians have fewer gastric parietal cells and poor metabolism of PPI [29]. Therefore, this study was conducted on almost the entire Korean population, using the exact date of prescription in the claims data and with adjustments for a large number of covariates. In addition, we analyzed the effects of any use, cumulative use, and regular use of PPI on the osteoporotic fracture risk.

There are several mechanisms by which PPIs increase the risk of osteoporotic fractures. First, PPIs reduce gastric acid by inhibiting H^+^/K^+^-ATPase at the last stage of gastric acid production. Calcium solubility plays an important role in calcium absorption, and the acidic environment of the gastrointestinal tract promotes the release of ionized calcium from insoluble calcium salts [33]. PPIs inhibit acid secretion and increase the pH, which slows down the dissolution of calcium salt and, consequently, prevents calcium absorption. The lack of calcium in the bone may increase the risk of osteoporotic fracture in the long term [34]. A second mechanism may be the reduction of vitamin B12 absorption induced by PPI [7]. The acidity of the stomach is important for the absorption of vitamin B12 [35]. It has been reported that inhibition of gastric acid by PPI may inhibit the absorption of vitamin B12 [36]. Vitamin B12 deficiency can cause homocysteinemia, leading to bone loss through increased osteoclastic activity, reduced osteoblast activity, reduced collagen linking, and decreased bone formation [37]. Vitamin B12 is also an essential co-factor for DNA synthesis and cellular energy production [38]. Continuous vitamin B12 deficiency can cause reversible megaloblastic aplastic anemia and demyelinated neuropathy leading to gait and weakness, which can cause neurological dysfunctions such as cognitive and visual impairment [39]. Therefore, vitamin B12 deficiency due to PPI use may be a risk factor for osteoporotic fractures. The third mechanism may be the increase in gastrin levels. Gastric acid is reduced by the use of PPIs, and the gastrin level is increased by the feedback mechanism. Hypergastrinemia due to PPI use has been reported to cause hyperplasia of the parathyroid gland, and increased parathyroid hormone could induce a decrease in bone mineral density [40, 41]. Also, PPIs interfere with the active transport of magnesium and can cause osteoporotic fractures [42].

### Strengths and limitations of this study

The first strength of this study is that we included a large number of patients using data from almost all Koreans. This study is also meaningful because it adds to the few Asian studies in the literature to date. Second, our study had a comparatively long observation period from 2006 to 2015. Third, the accuracy of drug use information over a long period is very high because the drug prescriptions were identified from NHIS claims data. Fourth, by using claims data and national health checkup data, various drugs and diseases affecting fracture could be used as covariates. In addition, the database allowed us to use accurate information without recall bias, which is common in retrospective studies.

Our study has some limitations. First, there was a possibility of residual confounding factors despite the attempt to adjust for factors influencing fracture. Due to the lack of data, we were unable to use lifestyle factors as covariates. The sources of residual confounding are also the lack of information regarding the disease severity and symptom severity of GERD and PUD, and the quality of care patients received in general by their healthcare providers. It is possible that healthcare providers using H2RA instead of PPI were more aware of the risk of PPI and were better educated regarding the management of geriatric patients. Second, we were not able to use medical records or imaging test results. Bone mineral density, a diagnostic parameter for osteoporosis, was also not available because this information is not included in the claims data. Third, our study outcome may include some fractures due to other causes, and may not include some osteoporotic fractures. The misclassification bias might not be eliminated.

## Conclusion

This nationwide nested case-control study demonstrated that osteoporotic fracture risk increased when PPIs were used for an extended period (≥ 1 year) or regularly both in men and women. The adverse effects of PPI on the risk of osteoporotic fractures were increased by increasing the duration of PPI use. Our findings suggest that reducing the duration of PPI use or avoiding their use altogether in favor of H2RA may reduce the risk of the occurrence of osteoporotic fractures in older adults. Appropriate benefits and risk assessments should be performed when prescribing PPIs. To shorten the duration of PPI prescriptions, clinicians may educate patients on lifestyle modifications to improve GERD symptoms or prescribe alternative medications with low gastric acid suppression. Also, especially when prescribing PPI to patients at high risk for osteoporosis, physicians may consider educating patients to prevent osteoporotic fractures.

## Data Availability

Data is available from the Korea National Health Insurance Sharing Service Institutional Data Access / Ethics Committee (https://nhiss.nhis.or.kr/bd/ay/bdaya001iv.do) for researchers who meet the criteria for access to confidential data. Researchers can apply for the National Health Insurance data sharing service upon approval of the Institutional Review Board of their institution. After review of the Korea National Health Insurance Sharing Service Institutional Data Access / Ethics Committee, authors are required to pay a data access fee and confirm that other researchers will be able to access the data in the same manner as the authors.

## Abbreviations

BMI: Body mass index
CI: Confidence interval
COPD: Chronic obstructive pulmonary disease
GERD: Gastroesophageal reflux disease
H2RA: Histamine-2 receptor antagonist
HRT: Hormone replacement therapy
OR: Odds ratio
PPI: Proton pump inhibitor
PUD: Peptic ulcer disease
SSRIs: Selective serotonin reuptake inhibitors
TCAs: Tricyclic antidepressants
